# Selection by higher‐order effects of salinity and bacteria on early life‐stages of Western Baltic spring‐spawning herring

**DOI:** 10.1111/eva.12477

**Published:** 2017-04-19

**Authors:** Maude Poirier, Luisa Listmann, Olivia Roth

**Affiliations:** ^1^ GEOMARHelmholtz Centre for Ocean ResearchKielGermany

**Keywords:** adaptation, *Clupea harengus*, common garden experiment, higher‐order effects, salinity, sex‐specific inheritance, *Vibrio spp*., Western Baltic spring‐spawning herring

## Abstract

Habitat stratification by abiotic and biotic factors initiates divergence of populations and leads to ecological speciation. In contrast to fully marine waters, the Baltic Sea is stratified by a salinity gradient that strongly affects fish physiology, distribution, diversity and virulence of important marine pathogens. Animals thus face the challenge to simultaneously adapt to the concurrent salinity and cope with the selection imposed by the changing pathogenic virulence. Western Baltic spring‐spawning herring (*Clupea harengus*) migrate to spawning grounds characterized by different salinities to which herring are supposedly adapted. We hypothesized that herring populations do not only have to cope with different salinity levels but that they are simultaneously exposed to higher‐order effects that accompany the shifts in salinity, that is induced pathogenicity of *Vibrio* bacteria in lower saline waters. To experimentally evaluate this, adults of two populations were caught in their spawning grounds and fully reciprocally crossed within and between populations. Larvae were reared at three salinity levels, representing the spawning ground salinity of each of the two populations, or Atlantic salinity conditions resembling the phylogenetic origin of *Clupea harengus*. In addition, larvae were exposed to a *Vibrio spp*. infection. Life‐history traits and gene expression analysis served as response variables. Herring seem adapted to Baltic Sea conditions and cope better with low saline waters. However, upon a bacterial infection, herring larvae suffer more when kept at lower salinities implying reduced resistance against *Vibrio* or higher *Vibrio* virulence. In the context of recent climate change with less saline marine waters in the Baltic Sea, such interactions may constitute key future stressors.

## 1 Introduction

Upon invasion of new habitats, genetic adaptation is expected for populations to survive and persist (Lee, Kiergaard, Gelembiuk, Eads, & Posav, 2011; Lee, Remfert, & Chang, 2007; Lee, Remfert, & Gelembiuk, 2003; Phillips & Shine, 2006; Reznick & Ghalambor, 2001), as natural environments are characterized by spatial and temporal variation of biotic and abiotic factors (Turner, 1989). Particularly strong selection on the inhabiting or invading populations acts in ecologically marginal environments (e.g., mountaintops, brackish‐water estuaries and arctic habitats; Johannesson & André, 2006). Bottlenecks are imposed on those animals living at the edge of their species distribution resulting in populations adapted to the extreme environmental conditions at the cost of a decreased genetic diversity (Johannesson & André, 2006). This adaptive differentiation can be driven by first‐order effects, i.e. physical variables such as light, temperature or salinity (reviewed by Sanford & Kelly, 2011). However, indirect or higher‐order effects, i.e. secondary consequences of primary variation, are becoming important when they shift the performance of interacting species. Here, pathogens are prime candidates that exert some of the strongest selection on hosts (Hamilton, Axelrod, & Tanese, 1990; Kawecki & Ebert, 2004). As often abiotic and biotic factors with interactive or even opposing impact structure a habitat, the outcome of the imposed selection is difficult to predict. A change in an abiotic factor may enhance pathogen virulence or decrease host tolerance (Schade, Raupach, & Wegner, 2016). Prevalent marine bacteria, i.e. *Vibrio* spp., are mostly opportunistic, but were suggested to enhance their growth (Kelly, 1982; Larsen, 1984) and virulence (Dayma et al., 2015; Wang & Chen, 2006) under decreased salinity and elevated temperature. In turn, animals were shown to decrease their immune response under low saline conditions (Birrer, Reusch, & Roth, 2012). Animals may thus not only be challenged to cope and adapt to the changed abiotic factors in the environment, but in addition suffer from more frequent and more harmful infections (freshwater example Briers, 2003; marine example Koprivnikar, Lim, Fu, & Brack, 2010). Thus, mechanisms that promote fast adaptation to novel environmental conditions are needed.

Epigenetic are not only involved in maternal effects but also in the nongenetic transfer of environmental experiences from fathers to offspring (Ashe & Whitelaw, 2007; Roth, Klein, Beemelmanns, Scharsack, & Reusch, 2012) with potentially strong impact on early life‐stages (Carr & Kaufman, 2009 **;** Rice et al., 1993; Rossiter, 1996). Parental experience could thus speed up offspring adaptive response to novel environmental conditions via a combination of epigenetics and sex‐specific inheritance. While paternal‐specific inheritance affects larval length in herring (Bang, Gronkjaer, Clemmesen, & Hoie, 2006) and hatching success in haddock (Rideout, Trippel, & Litvak, 2004) and Atlantic cod (Kroll, Peck, Butts, & Trippel, 2013), maternal‐specific inheritance impacts egg and larval size across various fish species (Chambers & Leggett, 1996; Heath & Blouw, 1998; Marteinsdottir & Begg, 2002).

A strong salinity gradient makes the Baltic Sea a highly stratified habitat that substantially differs from full marine waters such as the adjacent North Sea. The areas farthest from the ocean have lowest salinity and resemble freshwater environments. In the transition zone near the ocean, located in the Skagerrak and Kattegat, salinity is high and supports ecosystems of marine origin (Emeis, Struck, Blanz, Kohly, & Voss, 2003). As an almost land‐locked basin with only tight connection to the North Sea, the salinity of the Baltic Sea is influenced by the large river run‐off of freshwater (Segerstråle, 1969). The Baltic salinity gradient structures the habitat and imposes selection on genetic diversity and migration. It further promotes adaptation (Bonsdorff & Pearson, 1999; Laine, 2003; Westerbom, Kilpi, & Mustonen, 2002) resulting in a very low species‐diversity. Populations living in the Baltic Sea are, with few exceptions, both geographically and ecologically marginal, and some show clear evidence of being genetically deviant from North Sea and Atlantic populations of the same species (Luttikhuizen, Drent, & Baker, 2003; Väinölä & Hvilsom, 1991). As the freshwater lake that preceded the Baltic Sea opened to the North Sea about 8500 BP, freshwater periods alternated with more saline conditions (Russell, 1985). Most species found in the Baltic Sea are postglacial immigrants that live close to their salinity tolerance limits (Leppäkoski, Olenin, & Gollasch, 2002). While low salinity can hamper successful reproduction and development of marine fish (Nissling, Johansson, & Jacobsson, 2006), Baltic Sea populations compensated this negative effect with an adaptation to the lower salinity levels at the cost of a lower genetic diversity than Atlantic populations, as imposed by a tremendous bottleneck (Johannesson & André, 2006). Examples for adaptations to low salinity conditions include a shift in buoyancy level of cod eggs, which prevents them from sinking into oxygen‐depleted depths (Petereit, Hinrichsen, Franke, & Koster, 2014). In flounder, different gene expression patterns were found between Baltic and North Sea populations (Larsen et al., 2007). All these adaptive responses resulted in genetic differentiation: herring (Lamichhaney et al., 2012), turbot (Nielsen, Nielsen, Meldrup, & Hansen, 2004) and cod (Pocwierz‐Kotus et al., 2015) seem adapted to the respective salinity in their habitat.

The salinity levels may also correspond to particular bacterial compositions, as such adaptation of host immune defence against local bacteria in the pipefish *Syngnathus typhle* was identified (Roth, Keller, Landis, Salzburger, & Reusch, 2012). Acclimation to low salinity may thus be constrained by the additional challenge of high bacterial harm. *Vibrio spp*. is an omnipresent bacterium in coastal waters of the Baltic Sea and is known to cause diseases, like vibriosis, in fish populations (Colwell & Grimes, 1984). It was also found and isolated in Atlantic herring (*Clupea harengus*) (Bullock, 1977; Kennedy & Farrell, 2008), a key species in the Baltic Sea from an ecological and economical (fisheries) perspective. Atlantic herring invaded the Baltic Sea from the North Sea subsequent to the last glaciation, and since then underwent adaptation to low saline conditions (Johannesson & André, 2006). A subspecies of the Baltic herring, *Clupea harengus membras,* is even not capable of reproducing at oceanic salinity conditions anymore (Griffin et al., 1998).

Atlantic herring migrate from distinct spawning grounds to a common feeding ground and form seasonal aggregations of mixed populations (Ruzzante et al., 2006). In the Baltic Sea, herring populations seem genetically structured according to the salinity level in their spawning grounds, rather than to geographical distances (Bekkevold et al., 2005; Jorgensen, Hansen, Bekkevold, Ruzzante, & Loeschcke, 2005). This supports the assumption of natal homing in this species (Ruzzante et al., 2006). While gene flow among populations of different salinity seems rare (Gaggiotti et al., 2009), an empirical support for adaptation to salinity levels in spawning ground remains absent.

This study aimed to experimentally address adaptation to salinity and bacterial virulence at spawning grounds in two populations of Atlantic herring (*Clupea harengus*) spawning along the Baltic salinity gradient. Herring of two populations were crossed within and between populations. Larvae were subsequently exposed in a common garden approach to their native salinity, the salinity of the other population (novel salinity) and to a high salinity similar to North Sea conditions where herring originate from (original salinity). In addition, larvae were infected with *Vibrio* spp., one of the most abundant bacteria group in the marine environment. Life‐history traits and gene expression were evaluated to address genetic adaptation and the potential to cope with a shift in the interaction of abiotic (salinity) and biotic (*Vibrio* infection) factors. With the exposure to a high salinity, which resembles North Sea conditions, we aimed to assess how herring cope with the environmental condition of their phylogenetic origin. By comparing within‐ and between‐population crosses, the influence of sex‐specific inheritance and epigenetics for adaptation to adverse environmental conditions was assessed. We elucidated whether populations from less saline habitats are better in coping with a pathogen compared to populations from higher saline waters due to adaptation to enhanced virulence under low saline environmental conditions.

## 2 Methods

### 2.1 Experimental design

**Figure 1:**
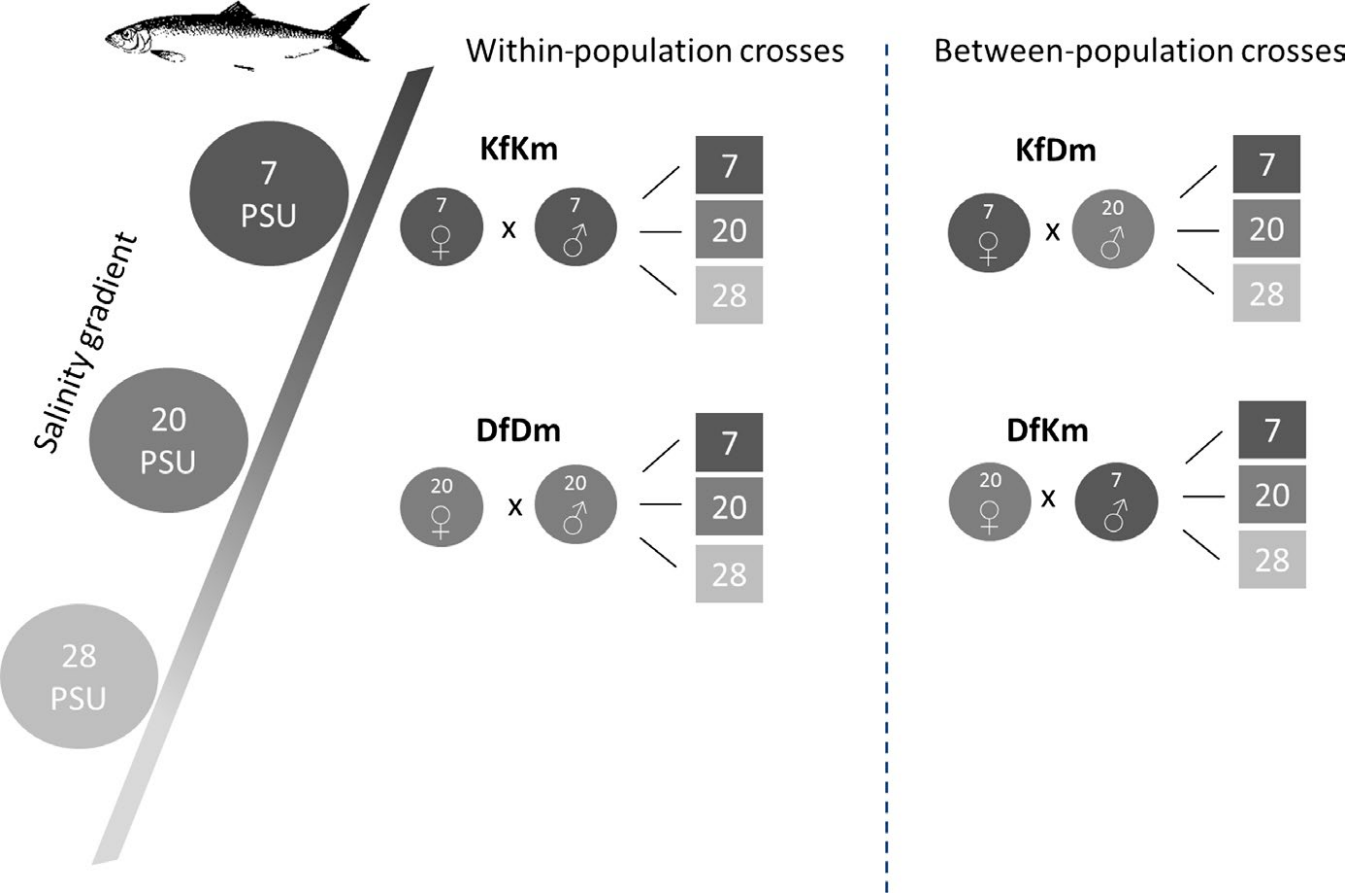
Experimental design. All four different crosses (KfKm, KfDm, DfDm, DfKm) are exposed to three salinity levels (7, 20 and 28 PSU), each treatment tank (square) is replicated eight times, *n* = 8. Abbreviations: K= Kiel, D= Danish, f= female, m= male

Two spawning areas of spring‐spawning herring in the Baltic Sea were selected for sampling. The spawning area in the Kiel Canal (Rade, N 54°20.368′/E 9°44.965′) has a low salinity level of approximately 7 PSU, whereas the spawning area in the Little Belt (Skaerbaek, Denmark N 55°30.781′/E 9°37.598′) shows a higher salinity level of approximately 20 PSU, located close to the Kattegat that builds the entrance of the Baltic Sea. The adult herring used in this experiment were caught by local fishermen mid‐April 2015. At both locations, eight ripe females and eight males were sampled, adding up to a total of 32 herrings. The gametes were stripped post‐mortem, directly at the corresponding location. The milt was collected in plastic beakers and the sticky eggs spread on plastic slides (11 × 5 cm) in two rows of one layer thick. Gametes were stored dry (undiluted) at 4°C on ice as suggested by Blaxter (1955). Due to logistical reasons, the whole sampling was performed on two consecutive days, starting in Denmark the first day and continuing at the Kiel Canal the next day. The gametes were crossed between and within both locations in a fully reciprocal design, resulting in four different crosses (Figure 1). From here on, the four crosses will be named as follow: KfKm, KfDm, DfKm and DfDm (K = Kiel, D = Danish, f = female, m = male). We exposed the fertilized eggs of each cross to three different salinity levels, 7 and 20 PSU correspond to the two study locations and 28 PSU served as the original salinity resembling North Sea conditions. The procedure of fertilization was conducted as follows: the sperm were activated by pouring seawater into the beaker and slightly swaying for a few seconds. Sperm were activated in the salinity level of the male's origin (Danish male at 20 PSU, Kiel Canal male at 7 PSU) to ensure an optimal activation. Always two slides with unfertilized eggs were put in tanks with seawater of the respective rearing salinity (7, 20 and 28 PSU). Fertilization was achieved by simply pouring the sperm solution into the tank. After 10 min fertilization was expected to be completed (Rosenthal, Klumpp, & Willfuhr, 1988) and the slides were put for another 10 min in a disinfection bath containing an Actomar solution (20 mL Actomar/1L saltwater) to minimize the risk of fungal infection. Each treatment tank was replicated eight times, resulting in 96 tanks (12 treatments × 8 replicates). One male and one female from each location were used for all four crossings of one replication level. The climate chamber was kept for the whole experiment at 8°C and low light (13 hr: 11 hr, light: dark). The rearing tanks (19 × 11 × 13 cm) were painted dark green from the outside to prevent the larvae swimming along the walls. The tanks were filled with 1.5 L saltwater and a 50% water exchange was done daily, as no flow‐through system was installed (Blaxter & Hempel, 1961). To achieve the three salinity levels, we used UV‐treated North Sea water at 28 PSU and diluted it with tap water to 20 and 7 PSU.

As a first life‐history trait, fertilization rate in each tank was estimated at day one and two postfertilization by dividing number of fertilized eggs by the total number of eggs. The second life‐history trait, hatching time, was measured by recording the hatching peak in days postfertilization for each tank. This time point is perceived as hatching time. All hatched larvae were counted to calculate hatching success (hatching success = Hatched larvae * 100/number of fertilized eggs). Four days after the relative hatching peaks, a bacterial infection (*Vibrio spp*.) was conducted with a subset of the larvae from each tank. The treatment was set at a time point where the larvae were still in the yolk‐sac stage, which implies that they had not switched to exogenous feeding yet but had already developed a mouth gap. This time point was chosen as *Vibrio spp*. infections are most likely invading the host via the gut (other possible entries are also gills and anus (Laurencin & Germon, 1987)). From each tank, forty larvae were transferred and divided into two plastic beakers (6 dl) filled with the corresponding salinity level (20 larvae per beaker). One beaker served as a control, and in the second beaker, *Vibrio spp*. was added. The *Vibrio spp*. treatment was conducted with a strain that was foreign to all populations. We are aware that with the use of sympatric *Vibrio* strains, we would likely obtain different results, as closely related *Vibrio* strains may cover a broad range of virulence (Wendling et al. 2017, in revision). However, our experimental design did not permit the testing of multiple *Vibrio* strains. We thus decided to limit our design to one strain that was foreign to all population to obtain comparable data for each population. This *Vibrio* strain was isolated in a previous study of Roth, Keller, et al. (2012) and Roth, Klein, et al. (2012) from pipefish *Syngnathus typhle* in Italy (strain I9K1). The *Vibrio spp*. strain was taken from a −80°C glycerol stock, suspended in a liquid medium (Medium 101: 5 g peptone + 3 g meat extract + 30 g NaCl in 1 L Millipore‐H_2_O, autoclaved and kept at room temperature) and grown in an overnight culture at 25°C. Thereafter, the bacteria solution was centrifuged for 10 min at 2,600 *g* and the supernatant removed. The bacteria pellet was resuspended in autoclaved seawater (7, 20 and 28 PSU) according to the salinity treatment in the beakers, yielding a concentration of 10^9^ bacteria cells per mL. Finally, 5 mL of the bacteria suspension was added to the *Vibrio* treatment beaker generating a concentration of 10^7^ cells per mL, respectively, and 5 mL of autoclaved seawater was added to the control beakers. 24 hr after the *Vibrio* treatment, 15 larvae from each beaker were sampled for gene expression analysis. Larvae were put directly in a RNA stabilizing reagent on ice (RNAlater^®^). Samples were then kept one day at 4°C and then stored at −20°C for further analysis. The five leftover larvae per beaker were used to address survival upon *Vibrio* exposure. Mortality was recorded daily until all larvae had died.

### 2.2 Genes of interest and primer design

Published and assembled herring transcriptome data were used to find suitable candidate genes for the gene expression analysis (Lamichhaney et al., 2012). A set of 32 genes was chosen involved in immune system, osmoregulation, epigenetic regulation, stress reaction and basic cell function using known sequences of stickleback and pipefish (Appendix S1: Table S1). The transcriptome was transformed to a blast database using the software BLAST (NCBI). Sequences (query) from all genes found on NCBI were blasted against the herring transcriptome to find matching sequences. Conserved regions and domains with functions were searched using Blastx and Blastn (NCBI) in the output sequences that were integrated into the primer design software (Primer 3 and NCBI Primer design tool). Primers flanking amplicons of a target size of 80‐200 bp were chosen. The transcriptome was used as a reference database to avoid multiple amplifications. Primer quality and efficiency were tested using quantitative real‐time PCR (RT‐qPCR; StepOnePlus^™^ Cycler, Applied Biosciences) by applying a cDNA dilution series. Primers with an efficiency range of >90% and a R^2^ value of >0.9 were considered (for detailed primer sequences see Appendix S1: Table S1).

### 2.3 Quantification of gene expression

For RNA extraction, three full‐sib larvae were pooled to ensure enough raw material for further gene expression analysis. RNA was extracted with an RNAeasy 96 Kit (Qiagen) according to manufacturer protocol. Extraction yields were measured by a spectrophotometer (NanoDrop ND‐1000; peQlab), and subsequently, 250 ng RNA was reverse transcribed into cDNA via QuantiTect^®^ Reverse transcription kit (Qiagen). Finally, 25 ng/μL cDNA was obtained and stored at −80°C. Expression of 32 genes was measured using Fluidigm‐BioMark^™^ system based on 96.96 dynamic arrays (GE‐chip). In a first step, a preamplification of the target cDNA was performed, containing 2.5 μL TaqMan PreAmp MasterMix (Applied Bioscience^®^) and 0.5 μL STA Primer mix (including all primer pairs 50 μM diluted in low EDTA‐TE buffer) under the following PCR conditions: 10 min at 95°C, 16 cycles: 15 s at 95°C, 4 min at 60°C. Thereafter, PCR products were diluted 1:20 with EDTA‐TE buffer. To load the chip, an assay mix of 0.7 μL of 50 μM primer pair mix, 3.5 μL 2xAssay loading reagent (Fluidigm) and 3.15 μL 1xlow EDTA‐TE buffer was prepared. The sample mix contained 3.3 μL preamplified cDNA, 3.5 μL 2xSsoFast EvaGreen Supermix with low Rox (BioRAD) and 0.35 μL 20xDNA Binding Dye Sample loading reagent (Fluidigm). After priming the chip with control line fluid, 5 μL assay mix and 5 μL sample mix were loaded on the chip and measured with Fluidigm‐BioMark^™^ system applying GE‐fast 96.96 PCR protocol according to Fluidigm instructions. Each chip contained no template controls (NTC) as well as gDNA contamination controls (‐RT). Samples were distributed randomly over the chips to control for technical bias and measured in technical triplicates.

### 2.4 Statistical analysis

All statistical analyses were performed in RStudio (R core team, 2015). The first part of the life‐history data (fertilization rate, hatching rate and time) was checked for normality using Shapiro–Wilk test and heterogeneity of variances using Levene's test. A three‐way ANOVA was performed to analyse main effects and interaction of the factors salinity level, maternal origin and paternal origin. A post‐hoc test (Tukey HSD) elucidated the significant differences between factor levels. The survival data contained no censored data, and survival rates were estimated using the Kaplan–Meier method according to the factors salinity level, maternal and paternal origin and *Vibrio* treatment. The median survival time of the five larvae per beaker was used to avoid the combination of pseudo‐ and biological replicates. The Cox proportional hazard model (coxph) was applied to identify differences among survival curves.

The gene expression data were accessed via the Fluidigm real‐time PCR analysis software (Fluidigm) to evaluate the amplification profiles. For the technical triplicates, mean Ct (cycle time), standard deviation (SD) and the coefficient of variation (CV) were calculated. CV gives an indication how precise a measurement is, and if CV is > 0.04, the value is falsified by a measurement error (Bookout & Mangelsdorf, 2003). Missing data points (2.4% of total data) and data points of CV> 0.04 (0.1% of total data) were replaced by the mean expression value of the affected gene (dummy variables). The most stable gene combination over all samples was analysed using the qbase+ software (Biogazelle). All analyses and plots are based on relative gene expression values (–δCt). Gene expression data were checked for normality using Shapiro–Wilk test and heterogeneity of variances using Levene's test. With an adjusted quantile plot, multivariate outliers were detected (Filzmoser, Ruiz‐Gazen, & Thomas‐Agnan, 2014) based on Mahalanobis distance. First a multivariate approach using PERMANOVA was applied to the whole data set (all 30 genes) and to the different gene groups (immune genes, epigenetic genes, osmoregulation genes, stress genes and cell function genes) to evaluate main effects and interactions of the four factors (salinity level, maternal and paternal origin and *Vibrio* treatment). For visualization, principal component analysis (PCA) plots were performed. Univariate analyses served to detect the single genes driving the effects identified in the multivariate analyses. Three‐way ANOVAs with type III sums of square, corrected for unbalanced design, were performed for each gene. Only factors with a significant effect in the multivariate approach are discussed. To elucidate the significant differences identified in the ANOVAs, post‐hoc tests (Tukey HSD) were applied.

## 3 Results

### 3.1 Fertilization rate

**Figure 2:**
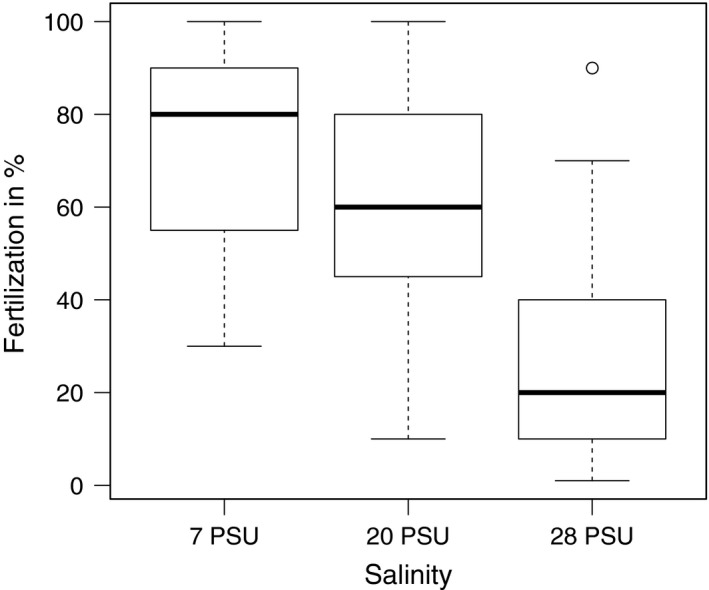
Effect of salinity (7, 20 and 28 PSU) on fertilization rate (box‐whisker plot)

**Figure 3:**
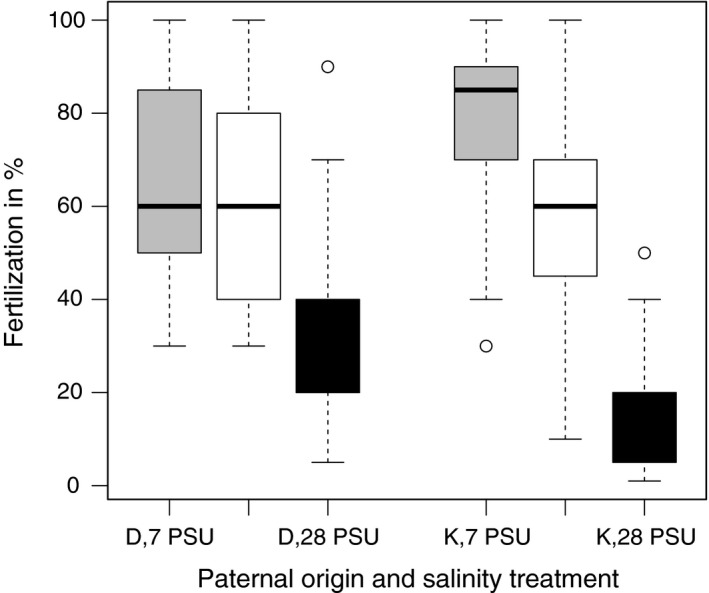
Interaction of paternal origin and salinity on fertilization rate (box‐whisker plot). 7 PSU (grey), 20 PSU (white) and 28 PSU (black). D=Danish male, K=Kiel male

Maternal origin (*p *<* *.01) and salinity (*p *<* *.001) as well as the interaction of paternal origin*salinity (*p *<* *.05) affected fertilization rate (ANOVA, Table 1). Fertilization rate decreased with increasing salinity (7 PSU: 72.9% ± 3.9 (mean ± *SEM*) > 20 PSU: 59.4% ± 4.01 > 28 PSU: 25% ± 4.1; Figure 2). Eggs from Kiel females showed a higher fertilization rate than eggs from Danish females (Kf: 59.9% ± 4.6 > *df*: 46.7%± 4.01). Sperm from Kiel males had the highest fertilization rate in their native salinity (7 PSU), whereas sperm from Danish males showed equal fertilization in 7 and 20 PSU, but lower fertilization in 28 PSU (Figure 3; for post‐hoc tests see Appendix S2: Table S1).

**Table 1:** ANOVA of salinity, maternal and paternal origin and *Vibrio* treatment on life‐history data (fertilization rate, hatching time and rate)

		Fertilization rate		Hatching time		Hatching rate	
ANOVAsFactor	Df	F value	*p*‐value	F value	*p*‐value	*F* value	*p*‐value
Maternal	1	9.092	.0034*	3.408	.0692	21.950	<.001*
Paternal	1	0.406	.5261	2.181	.1443	0.151	.699
Salinity	2	41.643	<.001*	62.522	<.001*	0.420	.659
Maternal*Paternal	1	0.396	.5309	2.544	.1153	0.415	.522
Maternal*Salinity	2	0.587	.5584	3.116	.0506	0.148	.862
Paternal*Salinity	2	4.659	.0123*	0.942	.3946	1.475	.235
Maternal*Paternal*Salinity	2	1.056	.3529	0.050	.9509	1.880	.16
Residuals		77		69		77	

*Denotes a significant result (*p *<* *.05).

### 3.2 Hatching time

**Figure 4:**
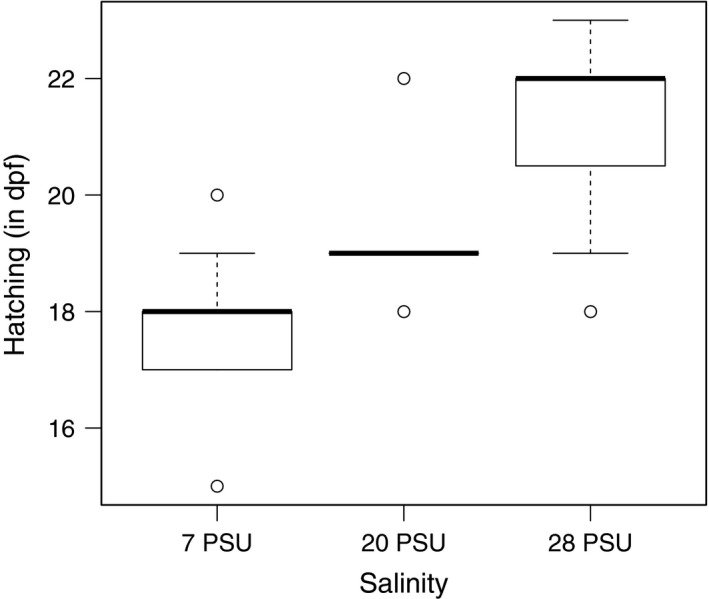
Effect of salinity (7, 20 and 28 PSU) on hatching time (box‐whisker plot). Hatching in days postfertilization (dpf)

Larvae hatched earlier at lower salinity (Table 1; salinity: *p *<* *.001; 7 PSU: 17.6 ± 0.17 (mean±*SEM*) days postfertilization (dpf) < 20 PSU: 19.4 ± 0.24 dpf < 28 PSU: 21.3 ± 0.29 dpf; Figure 4; for post‐hoc tests see Appendix S2: Table S2).

### 3.3 Hatching rate

**Figure 5:**
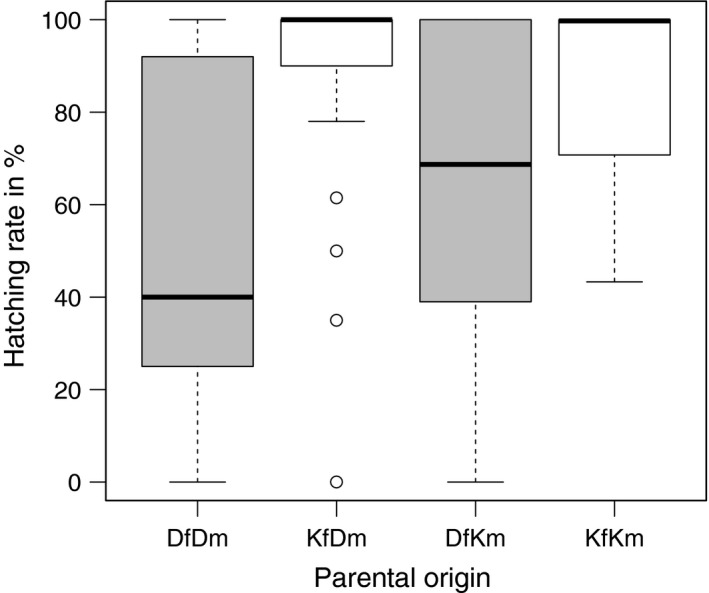
Effect of maternal origin on hatching rate in % (box‐whisker plot). Crossing with a Danish female (DfDm, DfKm) in grey, crossing with a Kiel female (KfKm, KfDm) in white

Maternal origin (*p *<* *.001) had an impact on hatching rate. The hatching rate was higher for crosses with a Kiel female (86.1%± 3.2) compared to crosses with a Danish female (55.5%± 5.6) (Table 1, Figure 5).

### 3.4 Mortality

**Figure 6:**
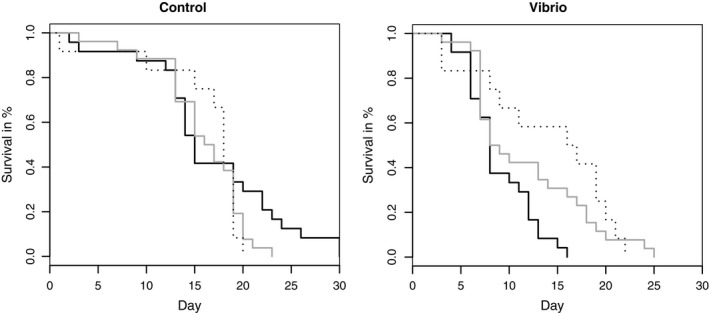
Salinity effect on median survival curves (Kaplan–Meier curve) in control (left panel) and *Vibrio* treatment (right panel); 7 PSU (black solid line), 20 PSU (grey solid line) and 28 PSU (dotted line). Survival in % (1.0 = 100%), Day 0 represents starting point of *Vibrio* treatment (equals 4 days postfertilization)

Vibrio exposure as well as salinity affected larval survival (proportional hazard likelihood ratio test, *Vibrio*:* p *<* *.001, salinity: *p *<* *.05). We identified a significant interaction of Vibrio*salinity (*p *<* *.001), maternal origin*salinity (*p *<* *.05) and paternal origin*salinity (*p *<* *.05), and in addition a significant three‐way interaction of maternal origin*salinity*Vibrio (*p *<* *.01; Table 2). If we only consider the abiotic and biotic main effect, we may conclude that larvae reared in 7 PSU lived longer than those reared at 28 PSU and that *Vibrio* infection shortened the lifespan of herring larvae (Appendix S2: Table S2). However, the interaction of those two factors gives insight into the importance of higher‐order effects: while larvae kept at 7 PSU suffer from a *Vibrio* infection, *Vibrio* had no impact at 20 PSU (Figure 6, Appendix S2: Table S2), and even enhanced survival at 28 PSU. In 20 PSU crosses with Danish males survived longer than crosses with Kiel males (Appendix S2: Fig. S1). In 20 PSU, larvae from crosses with a Kiel female coped better with the bacterial stress as larvae from Danish females (maternal origin*salinity*Vibrio). In 28 PSU, this pattern is reversed: larvae from crosses with Danish females coped better with both stressors than larvae from Kiel females (Appendix S2: Fig. S2, Table S2).

**Table 2:** Cox's proportional hazard fit of salinity, maternal and paternal origin and *Vibrio* treatment on mortality

		Survival analysis	
Proportional hazard likelihood ratio test Factor	*df*	Chi‐square	*p*‐value
Vibrio	1	18.755	<.001*
Maternal	1	0.818	.3659
Paternal	1	1.454	.2279
Salinity	2	6.721	.0347*
Maternal*Vibrio	1	0.017	.8952
Paternal*Vibrio	1	0.035	.8525
Maternal*Paternal	1	0.253	.615
Vibrio*Salinity	2	13.068	.0015*
Maternal*Salinity	2	8.732	.0127*
Paternal*Salinity	2	6.0281	.0491*
Maternal*Paternal*Vibrio	1	1.3445	.2462
Maternal*Salinity*Vibrio	2	9.6142	.0082*
Paternal*Salinity*Vibrio	2	2.4152	.2989
Maternal*Paternal*Salinity	2	2.2007	.3327
Maternal*Paternal*Salinity*Vibrio	2	0.8639	.6492

*Denotes a significant result (*p *<* *.05)

### 3.5 Gene expression

**Figure 7:**
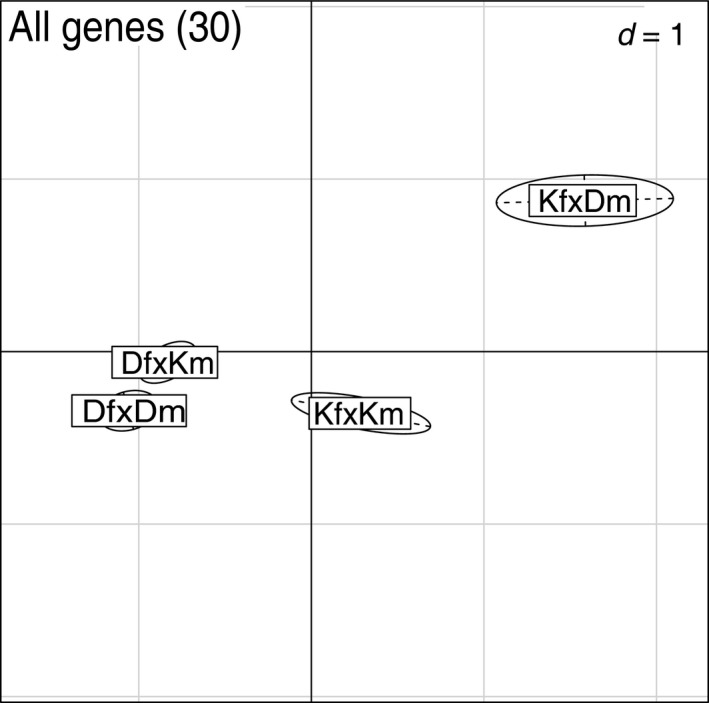
Principal component analysis (PCA) on all genes plotted by crossing. The first principal component (*x*‐axis) explains 44.12% of the variance, and the second principal component (*y*‐axis) explains 13.5% of the variances. Abbreviations: K = Kiel, D = Danish, f = female, m = male

**Figure 8:**
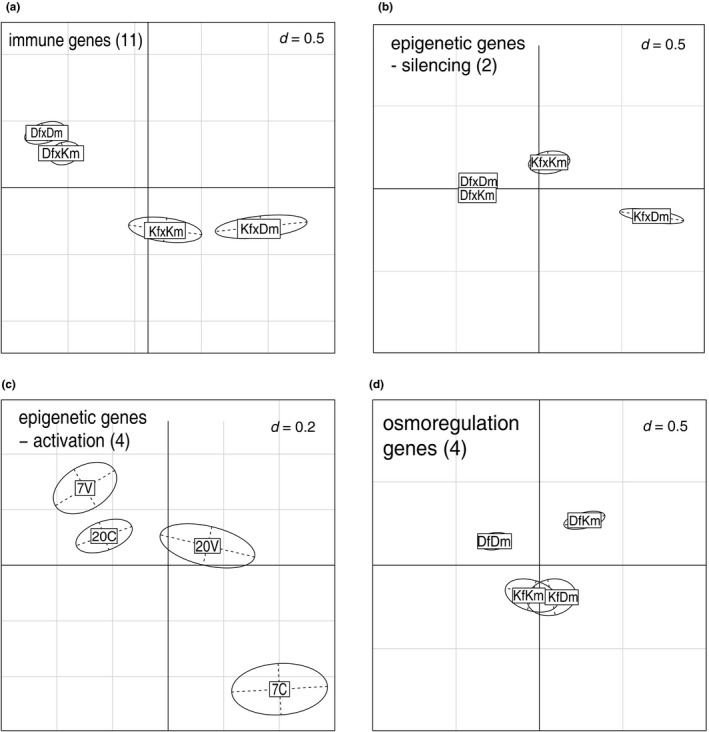
Principal component analysis (PCA): (a) immune genes, (b) epigenetic genes (silencing), (c) epigenetic genes (activation) and (d) osmoregulation genes plotted by crossing (plot a,b,d) and the interaction of salinity and Vibrio treatment (plot c). The first principal component (*x*‐axis) explains 44.12% of the variance, and the second principal component (*y*‐axis) explains 13.5% of the variances. Abbreviations: K = Kiel, D = Danish, f = female, m = male, V = Vibrio, C = control, 7 = 7 PSU, 20 = 20 PSU

With the geNorm algorithm of the Qbase+ software (Biogazelle), two epigenetic genes (silencing) RFD and SPRY were identified as most stable. They were thus used as reference genes throughout this study. Due to low hatching rate in 28 PSU and the loss of full tank replicates, the analysis of gene expression needed to be restricted to larvae kept at 7 and 20 PSU, with three to seven replicates for each treatment level (Appendix S3: Table S1), which corresponded to a total of 82 samples (six samples were detected as outliers and excluded according to Mahalanobis distance as described in the statistical analysis). The PERMANOVA over the whole data set (all 30 genes) yielded a significant main effect of maternal origin (*p *<* *.05) on gene expression (Table 3). Salinity and *Vibrio* as well as the higher‐order interaction showed no effect. From the principal component analysis (PCA, PC1: 44.12%, PC2: 13.5%), it can be depicted that DfDm and DfKm have an identical expression pattern, while both KfKm and KfDm have a different expression profile representing the impact of maternal origin (Figure 7). The PERMANOVA over all functional gene groups (immune genes, epigenetic genes, osmoregulation genes, stress genes, cell function genes) yielded significant main effects of maternal origin on “immune genes” (*p *<* *.01), “epigenetic genes‐silencing” (*p *<* *.05) and “cell function genes” (*p *<* *.05) as well as an interaction of Vibrio*salinity on “epigenetic genes activation” (*p *<* *.05) and an interaction of maternal*paternal origin on “osmoregulation genes” (*p *<* *.05) (Table 3). The PCA indicates for immune genes, epigenetic genes and osmoregulation genes a similar clustering of maternal origin as already described for the approach including all genes (Figure 8a,b,d), again larvae from Danish mothers were distinct from larvae from Kiel mothers, independent of the paternal origin. Larvae kept at 7 PSU that remained unexposed to *Vibrio* (7C) have a different expression profile of epigenetic genes (activation) than larvae exposed to *Vibrio* (7V and 20V) and control larvae kept at 20 PSU (20C) (salinity*Vibrio) (Figure 8c).

**Table 3:** PERMANOVA of salinity, maternal and paternal origin and *Vibrio* effect on gene expression in –δCt of all genes combined and individual gene groups

PERMANOVAs Factor	*df*	all genes (30)		immune (11)		epigen‐activ (4)		epigen‐silen (2)		osmo genes (4)		basic (3)	
		R^2^	*p*‐value	*R* ^2^	*p*‐value	*R* ^2^	*p*‐value	*R* ^*2*^	*p*‐value	R^2^	*p*‐value	R^2^	*p*‐value
M aternal	1	.038	.016*	.064	.002*	.031	.086	.058	.027*	.004	.673	.047	.030*
Paternal	1	.016	.24	.004	.871	.007	.585	.026	.128	.028	.113	.012	.389
Vibrio	1	.008	.646	.005	.855	.007	.562	.005	.567	.017	.211	.003	.779
Salinity	1	.011	.465	.016	.285	.002	.924	.007	.482	.021	.179	.001	.916
Maternal*Paternal	1	.022	.121	.012	.381	.011	.397	.025	.141	.049	.034*	.014	.315
Maternal*Vibrio	1	.004	.959	.003	.913	.005	.729	.004	.614	.001	.951	.007	.536
Paternal*Vibrio	1	.003	.994	.003	.972	.010	.452	.002	.761	.001	.915	.002	.907
Maternal*Salinity	1	.007	.764	.006	.728	.010	.42	.006	.465	.001	.935	.011	.406
Paternal*Salinity	1	.013	.36	.006	.775	.002	.926	.007	.08	.024	.175	.015	.296
Vibrio*Salinity	1	.016	.225	.018	.191	.051	.014*	.038	.45	.002	.789	.012	.377
Maternal*Paternal*Vibrio	1	.003	.979	.006	.747	.003	.857	.005	.569	.002	.847	.002	.862
Maternal*Paternal*Salinity	1	.006	.776	.011	.428	.001	.96	.007	.455	.002	.896	.003	.845
Maternal*Salinity*Vibrio	1	.009	.521	.009	.572	.022	.143	.020	.204	.007	.482	.003	.78
Paternal*Salinity*Vibrio	1	.014	.31	.019	.191	.034	.064	.007	.462	.001	.881	.014	.293
Maternal*Paternal*Salinity*Vibrio	1	.009	.576	.008	.589	.008	.536	.012	.352	.002	.872	.019	.214
Residuals	66												
Total	81												

The group of “stress genes” is not shown, as it contains no significant result. Number in brackets indicates number of genes contained in that group. *denotes a significant result (*p *<* *.05).

The univariate analyses identified that the expression of eight genes drives the effects in the functional gene groups (ANOVA). The immune gene CC3 was downregulated in crosses with a Danish female, whereas the immune genes IK cytokine and integrin‐beta 1 were upregulated in crosses with a Danish female compared to crosses with a Kiel female (Appendix S3: Fig. S1, Table S2). Both epigenetic genes (silencing) ADDZ and HDAC1 were upregulated in crosses with a Danish female compared to crosses with a Kiel female (Appendix S3: Fig. S1, Table S2). The cell function gene EF1‐alpha was upregulated in crosses with a Danish female compared to those with a Kiel female. And for the osmoregulation gene NHE1, an interaction of paternal*maternal origin was found, where larvae from KfKm showed an upregulation compared to KfDm (Appendix S3: Fig. S2, Table S2, S3). Moreover, a significant interaction of salinity*Vibrio was found on the expression of the epigenetic gene TPR (*p *<* *.01), with an upregulation in *Vibrio*, 7 PSU and Control, 20 PSU treatment and a downregulation in Control, 7 PSU and *Vibrio*, 20 PSU treatment (Appendix S3: Fig. S3, Table S2, S3).

## 4 Discussion

The Western Baltic spring‐spawning herring reproduces in areas with different salinity levels and is able to cope with low saline conditions. We identified an induced performance of larvae from two Baltic Sea herring populations in low saline waters (resembling Baltic Sea conditions) compared to high saline waters (resembling North Sea conditions). However, induced bacterial virulence at lower salinity levels may partly outweigh benefits of adaptation to low saline waters.

By comparing parental fertilization success, hatching and performance of herring larvae in their native versus novel salinity, we aimed to assess selection as one prerequisite for adaptation. Sex‐specific inheritance influenced fertilization rate. Crosses with a Kiel male had the highest fertilization rate in their native salinity (7 PSU), but for crosses with a Danish male, the fertilization rate was equal in native (20 PSU) and in the novel salinity (7 PSU). The hydrological features defining the two locations may answer why the fertilization optimum of Danish sperm is wider compared to Kiel sperm. The Kiel Canal is an artificially enclosed water body with a stable salinity regime (salinity range from west to east: 5.5–11.5 PSU (Paulsen, Clemmesen, Hammer, Polte, & Malzahn, 2016). The Little Belt, in contrast, renders larger amplitude of salinity fluctuations induced by the inflow of North Sea water and the impact of wind (annual variation from 14–23 PSU; Conley, Kaas, Mohlenberg, Rasmussen, & Windolf, 2000). Stable environments are supposed to select for genetic adaptation, while fluctuating environments may rather select for phenotypic plasticity (Lande, 2009). While both populations are consistent with adaptation to salinities occurring in the Baltic Sea (note that for a final conclusion about genetic adaptation, the parental generation would also need to be examined), the Kiel Canal population may in addition be in a process of local adaptation to spawning ground salinity. The potential of local adaptation to salinity in Atlantic herring of the Western Baltic was suggested by a study with genetic markers (Bekkevold et al., 2005; Gaggiotti et al., 2009). In accordance with this, larvae from crosses with Danish males survived better in their native salinity (20 PSU) than larvae from Kiel males in that same salinity. Surprisingly, the weak indicators of potentially ongoing local adaptation depended on the paternal but not on the maternal origin.

The original salinity level (North Sea salinity, 28 PSU) was used throughout the experiment to assess how the two populations cope with the salinity conditions of their genetic origin. We found no population‐specific responses to the original salinity. But all crosses suffered at 28 PSU as indicated by lowest fertilization rate at 28 PSU, delayed hatching time and reduced survival. The delay in hatching is consistent with the study from Griffin et al. (1998). They investigated hatching time in Pacific herring (*Clupea pallasi*) and found a delay at the highest salinity. The reason for this delay could lie in a resource allocation trade‐off, as more energy is needed to maintain the ionic balance, and therefore, less energy is available for embryo development (Griffin et al., 1998). In this study, most tanks were lost at 28 PSU (eight tanks out of 96 tanks), as fertilization was zero or full batches of larvae did not hatch. The larvae that hatched at this high salinity died earlier (Fig. 6, left). All these findings indicate an approach of the effective upper salinity tolerance limit for embryo development of Western Baltic herring. Colonization of fishes from the North Sea to the Baltic Sea is thought to have occurred mainly during the Littorina stage (7,500‐4,000 years ago) (Ojaveer & Kalejs, 2005; Segerstråle, 1969). Therefore, 28 PSU can be seen as the ancient salinity level of herring stocks living in the Baltic Sea. Our findings indicate that stocks of Atlantic herring occurring nowadays in the Baltic Sea (here Western Baltic spring‐spawning herring) adapted to the lower Baltic salinity ranges in several early life‐history traits.

Hatching rate was driven by the maternal origin: more larvae hatched from crosses with a Kiel mother (within‐ and between‐population crosses) than from crosses with a Danish mother. While Kiel Canal eggs may in general have a higher hatching rate than Danish eggs, hatching rate could also be confounded by the experimental design. Kiel eggs were collected 24 hr later than Danish eggs and may therefore be fresher and of better quality; however, according to Blaxter (1955), eggs and sperms should hold quality up to 48 hr after collection (when stored dry at 4°C) making a confounding effect during the first 24 hr of storage unlikely. The same pattern was also identified for the fertilization rate, as eggs from Kiel females showed a higher fertilization rate than Danish eggs. Maternal origin also affected the expression of genes as depicted from the PERMANOVA over all genes, showing a distinct expression pattern for larvae with a Kiel mother (KfKm and KfDm) in contrast to larvae with a Danish mother (DfDm and DfKm). This effect was further identified for several immune genes and two epigenetic genes involved in silencing. The differences in immune gene expression of larvae depending on maternal origin may suggest that animals originating from the two locations vary in their parental pathogenic experience. The transfer of phenotypic plastic pathogenic experience and immunological information via the egg or via epigenetic changes could influence offspring gene expression patterns (Magnadottir, Lange, Gudmundsdottir, Bogwald, & Dalmo, 2005).

With the exposure of larvae to a bacterial challenge, we aimed to elucidate three main questions: can we find higher‐order effects of interacting abiotic (salinity) and biotic (*Vibrio*) factors; does parental origin matter in the ability to cope with a pathogenic *Vibrio* bacteria: and are *Vibrio* bacteria less harmful at low salinity in populations spawning at lower salinity level (e.g., Kiel Canal, 7 PSU). Answering this, we can elucidate whether adaptation towards low salinity is accompanied by resistance evolution against potentially more virulent infections at lower salinities. Mortality was influenced by an interaction between *Vibrio* and salinity. We found a higher mortality of *Vibrio spp*. challenged larvae in 7 PSU than in 28 PSU. As the larval immune gene expression was not directly influenced by the *Vibrio* infection as demonstrated in other studies (Birrer et al., 2012), the high mortality of larvae seems to be caused by an enhanced virulence of the chosen bacteria strain in lower salinity. At 28 PSU and 20 PSU, *Vibrio spp*. was avirulent, as determined by an equal survival between control and *Vibrio* treatment at 20 PSU, and an even increased survival at 28 PSU if larvae were exposed to *Vibrio*. In the perspective of climate change, the predicted drop in salinity implies an additional biotic stressor for marine organisms: opportunistic *Vibrio* bacteria are becoming harmful at low salinities. The interaction of salinity and *Vibrio* further affected the expression of epigenetic genes (activation), where larvae in 7 PSU unexposed to bacteria (7C) showed a distinct expression profile than the treatments with bacteria and higher salinity (20C, 20V and 7V). This indicates that epigenetic factors play a role in handling salinity and *Vibrio* stress, consistent with their function in buffering environmental change (Bonduriansky, Crean, & Day, 2012). The epigenetic marks could modify the phenotypic plasticity of the offspring and once these marks become genetically assimilated, they may fasten the adaptation of populations towards abiotic and biotic environmental factors (Jablonka & Lamb, 1998, 2015).

While in low saline waters (7 PSU), all crossings survived longest without an exposure to prevailing bacteria, the opposite pattern was found upon application of *Vibrio* infections. This implies that *Vibrio* infections were most harmful in low saline waters. At higher salinities, mortality was rather influenced by a higher‐order salinity*Vibrio interaction that depended on the fish origin. Under a *Vibrio* infection, larvae from Kiel mothers lived longer in 20 PSU than larvae from Danish mothers; at 28 PSU, the pattern was reversed. Hence, only offspring from a Danish mother could cope with the dual stress imposed by salinity change and *Vibrio* infection, once the salinity reached the original North Sea levels (28 PSU). This implies that the genetic background matters in the ability to cope with the higher‐order environmental factor (Vibrio*salinity) and may even go in line with the prediction that Danish population could genetically be closer related to the original North Sea populations, as the imposed bottleneck was potentially less severe (Johannesson & André, 2006). This pattern was not revealed in immune gene expression, potentially due to a limited repertoire of candidate immune genes. In addition, gene expression was only assessed for crossings at 7 and 20 PSU. Alternatively, the 24 hr between exposure and sampling might have not been sufficient to induce an immune defence, as the larvae were immunologically naïve against the applied bacteria strain. The composition of *Vibrio* phylotypes in the Baltic Sea is influenced by abiotic factors such as temperature, salinity and phosphorus concentration (Eiler, Johansson, & Bertilsson, 2006). While *Vibrio alginolyticus* has a higher abundance in high saline waters, *Vibrio anguillarum* shows the opposite pattern (Eiler et al., 2006). Herring adults most likely have to raise an immune response against a high *Vibrio* diversity that they experience during their migratory phase. This can result in a diversified immune competence and immunological memory of adult herring that may even be transferred via mothers to the offspring. The *Vibrio* isolate used in this study was a strain from Italy and supposed to be allopatric for both populations, such that none of the herring adults were expected to have experienced this strain during their lifespan.

Our data are consistent with an adaptation of Western Baltic spring‐spawning herring towards the low salinity level in the Baltic Sea. Moreover, we detected a sex‐specific driver (paternal origin) for the traits that may indicate ongoing local adaptation. The increased *Vibrio* harm under lower salinity seems to impose stronger selection on herring early life‐stages than the salinity itself. Higher‐order biotic effects like the here observed bacterial pathogenicity are thus prone to act as “amplifiers” of a rapidly changing marine environment, where among many other abiotic factors such as CO_2_ partial pressure and temperature, also freshwater impact and, hence, salinities are predicted to change also on a global scale.

## Animal Welfare

The animal welfare officer of the University of Kiel confirmed that, according to German animal welfare act, this study does not need an ethic approval. Investigations such as collecting eggs and sperm from adult herring that were commercially caught by fisherman, and growing larvae up to the first feeding stage are legal without an animal welfare permit. Therefore, these studies are not investigations in which animals suffer and thus they are no subjects of the German animal welfare act.

## Data Archiving Statement

Data for this study are available at Pangaea: https://doi.pangaea.de/10.1594/PANGAEA.873085.

## Author Contribution

MP and OR designed and performed the experiment, laboratory work, statistical analysis and wrote the manuscript; LL designed the primers (except the epigenetic primer, which were designed by MP).
