# Supplementary material for: Selection by higher‐order effects of salinity and bacteria on early life‐stages of Western Baltic spring‐spawning herring
Source: Evol Appl. 2017 Apr 19;10(6):603–15. doi: 10.1111/eva.12477 (PMC5469169; doi:10.1111/eva.12477)
Supplement: Supplementary file 1 [file EVA-10-603-s001.docx]

**Supplementary Material**

Selection by higher-order effects of salinity and bacteria on early life-stages of Western Baltic spring-spawning herring

Maude Poirier^1^, Luisa Listmann^1^ and Olivia Roth^1^

^1^GEOMAR, Helmholtz Centre for Ocean Research Kiel, Düsternbrookerweg 20, 24105 Kiel, Germany

Email-address:

mpoirier@geomar.de,

llistmann@geomar.de,

oroth@geomar.de (corresponding author)

**Appendix 1: Genes of interest and primer sequences**

**Table S1** Genes analysed for their expression profile. Genes are sorted in six groups according their functions in the organism (epigenetic genes, cell function genes, immune genes, osmoregulation genes and stress genes). Primer sequences and amplicon length are shown as well as primer efficiency assay (efficiency in % and R^2^).

| Group | Gene | Abbrev | Fwd (5´→3´) | Rev (5´→3´) | Amp (bp) | R^2^ | Eff (%) | Function |
| --- | --- | --- | --- | --- | --- | --- | --- | --- |
| Basic cell function  genes | Beta-actin | β-actin | GGTGTGATGGTCGGTATGGG | CTCATTGTAGAAGGTGTGATGCC | 156 | 1 | 94.3 | Cell motility, cytoskeleton |
|  | Elongation Factor 1 alpha | EF1α | TTCAAGTACGCTTGGGTGCT | GCAACAATCAGCACAGCACA | 191 | 0.99 | 95.4 | Transport of tRNAs to ribosome |
|  | 18S ribosomal RNA | 18SrRNA | ACAGTGAAACTGCGAATGGC | GCATGTATTAGCTCTGGAATTGCC | 99 | 1 | 101.8 | Protein synthesis |
|  | Apolipoprotein E | Apolip E | GAACCCGGACTTCTCAGGTG | CGCTCCACACTTTCTTCCCA | 145 | 1.000 | 98.2 | Lipid metabolism |
| Osmoregulation genes | V-type-H^+^-ATPase subunit A | V-H^+^-ATPase | TCCAGCGTCCACTGAAAGAC | GATATGGCTTCCGACTCGCA | 137 | 0.96 | 98.6 | Osmoregulation, ion exchange  (Varsamos 2010) |
|  | Na^+^-K^+^-ATPase | ATN-A1 I | TCCTGTGGCTGTGTGAGAAC | ATGGACGGAGAGCTGGTACT | 93 | 0.99 | 94.5 | Osmoregulation, ion exchange (Varsamos 2010) |
|  | Na^+^/H^+^ exchanger 1 | NHE1 | GTGTGCTTCCTGGTGGTGTC | AAGACGAAGAGGGGCTCGAT | 119 | 0.99 | 94.2 | Maintaining of ion homeostasis (Vilella 1995) |
|  | Na^+^/HCO_3_^-^ cotransporter | NBC1 | AGCAGCCTAAACGACCTGTC | GTCAGACCTCCCAGCATGAC | 179 | 0.98 | 95.5 | Osmoregulation (Taylor 2010) |
| Stress genes | Heat shock protein Dnaj4 | hspDnaj4 | AGAGGCGTACAGATCCAGGT | CCAGTGCAGTTCTTGCATCG | 128 | 0.998 | 90.4 | Stress-induced response, chaperone |
|  | Heat shock protein 70 | hsp70 | AAGGCAGTTACGCATTTCGG | GAAAAGCCCGTAAGTCATGGG | 97 | 1.000 | 97.0 | Stress-induced response, chaperone |
|  | Heat shock factor 1 | hsf1 | TGCGACAGTTGTTTTCTACGC | CCAAGAAACCTTGACAGAATCCG | 194 | 0.992 | 92.6 | Stress-induced response, chaperone |
|  | Heat shock protein 90 | hsp90 | GGTGGACTCTGAGGATTTGCC | CACTTCTTGACCAGATTCTTGCG | 96 | 1.000 | 95.0 | Stress-induced response, chaperone |
|  | Growth arrest and DNA damage inducible protein 45 alpha | GADDIP45α | ATACGAGGCGGCAAAGTCTC | CTCACAGCAGAATGCCTGGA | 133 | 0.999 | 92.1 | Activated by DNA damage |
| Immune genes | Gene associated with retinoic-interferon-induced mortality 19 | GRIM19 | CAAGGTGAAGCAGGACATGC | TAACCCCGATGCCAATACCG | 116 | 1 | 95.7 | Innate immunity, cell death regulation |
|  | Natural resistance associated macrophage protein | NRAMP | AACGACTTCCTCAACGTGCT | CGCCGCCAATCTTCCAAAAT | 127 | 0.99 | 102.0 | Innate immunity, iron transport |
|  | IK cytokine | IK CK | AGGTACGAGCCGAGATCACT | ACTGTCGGCCTTTCATCAGG | 159 | 0.99 | 92.7 | Adaptive and innate immunity, signalling protein |
|  | Kinesin Family mem. 13b | KFM 13b | AGCTCTACCAGGAGGGGAAG | CCACCCCGATTAGACTGTGG | 114 | 0.99 | 95.9 | Innate immunity |
|  | Integrin beta 1 | Iβ1 | CCCACCTGGTACAGAAGCTG | GAGGAGAGTGTTCCCACTGC | 127 | 1 | 90.8 | Adaptive immunity; cell adhesion |
|  | Natural killer enhancing factor | NKEF | GTGCGCTTCAAAGGAGTCTG | CCCTCTGTAGTCGGAGAGCT | 185 | 1 | 89.8 | Innate immunity |
|  | Akirin | Akr | TAGCAAACATGGCGTGTGGA | CCGGTAATGGACTGCACCTT | 102 | 0.99 | 96.5 | Innate immunity, downstream effector of the Toll-like receptor |
|  | Tumor necrosis factor 2 | TNF2 | CTGGTGCTGGTGGAGAACTT | CAGCTCACGACACTCGTTGA | 129 | 1 | 93.4 | Innate immunity, inflammation response |
|  | Translocator protein | TSPO | AGTTAGCCGCACCCATTCTT | GGGCTGTGAGACCCAACATT | 153 | 0.96 | 95.4 | Innate immunity , inflammation response |
|  | Complement component 1 Q subcomponent-binding | CC1Qsub | AGGGCAGTGCTTAATTCCGT | GGGACACGGACTGGAAAGAA | 145 | 0.99 | 100.8 | Innate immunity, complement system |
|  | Complement component C3 | CC3 | AGCGGCTGACATAGGCATTT | GGCCGAATACAGGAGATGCA | 125 | 0.91 | 97.5 | Innate immunity, complement system |
| Epigenetic genes | Histone-acetyltransferase KAT2A (Bromodomain) | BROMO | TGGAACCAGTAAAGAAGTCCGA | TGGTCACATAGTAGCGGTTCTT | 108 | 0.903 | 111.7 | Acetylation of histones at lysine residues (gene activation) |
|  | Histone-acetyltransferase KAT2A (PCAFdomain) | PCAF | CCAGACCATGTTTGAGCTGAGT | ATGTGGAGCCGTCATCTTTCTG | 110 | 0.881 | 93.8 | Acetylation of histones, predominantly helical  (gene activation) |
|  | Histone-acetyltransferase KAT8(MOZ/SASdomain) | MOZ | TGGACAACGTTCCCCTGAAG | CAGAGAAGCCGCTGTCAGAT | 99 | 0.993 | 123.5 | Acetylation of histones  (gene activation) |
|  | Histone-deacetylase 1B (HDAC1domain) | HDAC1 | GATGGCATTGACGACGAATCTT | ACGGCAGGTTAAAGCTCTTCAT | 196 | 0.960 | 98 | Deacetylation of histone at lysine residues, involved in DNA-damage response (gene silencing) |
|  | Histone-methyltransferase (SPRYdomain Ash2) | SPRY | GATTACGTGGACAAGGCAGAGA | TGGCTCACACCGTTCTTGTAG | 80 | 0.890 | 96.7 | Methylation of histones, Ash2 protein involved in transcriptional regulators of Hox genes (gene silencing) |
|  | Lysine-specific demethylase_6A (TPRdomain) | TPR | GTAAACCATACCGAGGCCGT | AAGGCTGTTCGCTGCTATGA | 198 | 0.970 | 108 | Demethylation of histones  (gene activation) |
|  | DNA(cytosine-5)-methyltransferase1 (RFDdomain) | RFD | ATGAGGCCCGAGTCAAATGG | GAGCTGCCTCAGCACAAAAT | 80 | 0.950 | 107.5 | Methylation of cytosine, involved in DNA repair and genome stability (gene silencing) |
|  | DNA(cytosine-5)-methyltransferase3A (ADDZdoamin) | ADDZ | TCAGTATGACGACGATGGCTAC | ACCCACTAACAGATCCACACAC | 127 | 0.96 | 93.8 | Methylation of cytosine, inheritance of pattern during mitosis (gene silencing) |

**Appendix 2: Post-hoc on life-history data**

Table S1 Post hoc analysis (Tukey’s test) of treatment factors on life-history traits (fertilization rate and hatching time). Abbreviations: Km= Kiel male, Dm=Danish male; * denotes a significant result (p<0.05).

| Post hoc test |  |
| --- | --- |
| Fertilization rate | **p-value** |
| 7PSU- 20 PSU | **0.029*** |
| 7 PSU- 28 PSU | **0.00***** |
| 20 PSU- 28 PSU | **0.00***** |
| Km, 7 PSU- Km, 20 PSU | **0.0359** |
| Km, 7 PSU- Km, 28 PSU | **0.00***** |
| Km, 20 PSU- Km, 28 PSU | **0.00***** |
| Dm, 7 PSU- Dm , 20 PSU | 0.9928 |
| Dm , 7 PSU- Dm , 28 PSU | **0.0018**** |
| Dm , 20 PSU- Dm , 28 PSU | **0.0088**** |
| Km, 7 PSU- Dm, 7 PSU | 0.4235 |
| Km, 7 PSU- Dm, 20 PSU | 0.1412 |
| Km, 7 PSU- Dm, 28 PSU | **0.00***** |
| Km, 20 PSU- Dm, 7 PSU | 0.8582 |
| Km, 20 PSU- Dm, 20 PSU | 0.9906 |
| Km, 20 PSU- Dm, 28 PSU | 0.0518 |
| Km, 28 PSU- Dm, 7 PSU | **0.00***** |
| Km, 28 PSU- Dm, 20 PSU | **0.00***** |
| Km, 28 PSU- Dm, 28 PSU | 0.1760 |
| Hatching time | **p-value** |
| 7PSU- 20 PSU | **2.00e-07***** |
| 7 PSU- 28 PSU | **0.00***** |
| 20 PSU- 28 PSU | **6.7e-06***** |

**Table S2** Parameter estimates of Cox’s proportional hazard fit on mortality.

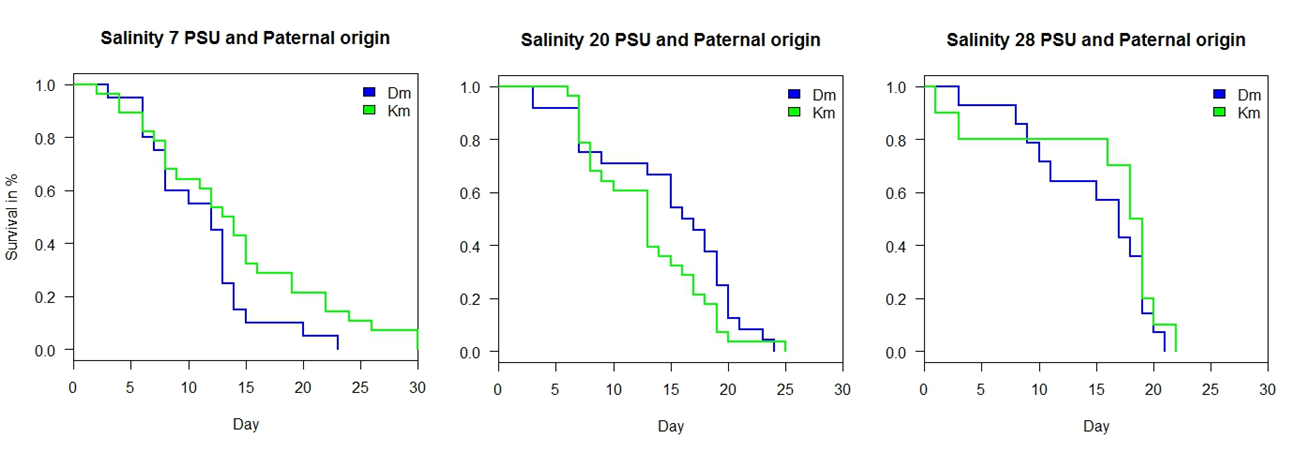


Fig. S1 Interaction of paternal origin and salinity on survival. Survival in % (1.0 = 100%), curves represents median survival per beaker.


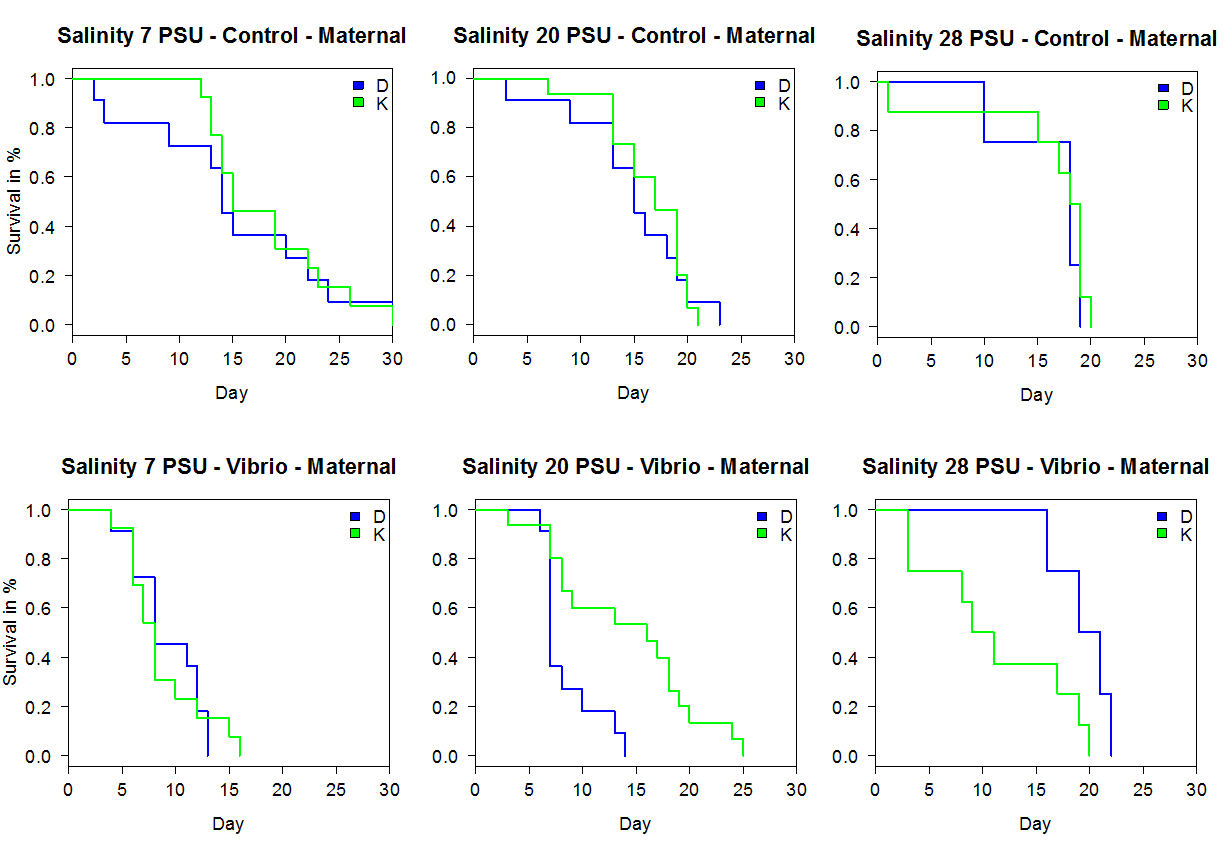


Fig. S2 Interaction of maternal origin, salinity and *Vibrio* treatment on survival. Survival in % (1.0 = 100%), curves represents median survival per beaker

**Appendix 3: univariate analysis on gene expression data**

**Table S1** Number of replicates of each treatment group used for gene expression analysis. Abbreviations: K= Kiel, D= Danish, f= female, m= male

| **Control** | | | **Vibrio** | |
| --- | --- | --- | --- | --- |
|  | **7 PSU** | **20 PSU** | **7 PSU** | **20 PSU** |
| DfDm | 7 | 3 | 6 | 4 |
| DfKm | 5 | 4 | 5 | 5 |
| KfDm | 4 | 7 | 4 | 4 |
| KfKm | 5 | 7 | 6 | 6 |

**Table S2** Univariate statistical analysis (ANOVA) on gene expression of single target genes from gene groups with a significant result. * denotes a significant result (p<0.05).

**Table S3** Post hoc analysis (Tukey’s test) on gene expression of single genes. * denotes a significant result (p<0.05).

| Post hoc test |  |
| --- | --- |
| TPR | **p-value** |
| 7 PSU, C - 20 PSU, C | 0.1664 |
| 7 PSU, V - 7 PSU, C | 0.1341 |
| 7 PSU, C - 20 PSU, V | 0.9882 |
| 7 PSU, V – 20 PSU, C | 0.9995 |
| 20 PSU, V - 20 PSU, C | 0.3245 |
| 7 PSU, V - 20 PSU, V | 0.2740 |
| NHE1 | **p-value** |
| KfDm – DfDm | 0.2354 |
| DfKm – DfDm | 0.9691 |
| KfKm – DfDm | 0.8835 |
| DfKm – KfDm | 0.4827 |
| KfKm – KfDm | **0.0407** |
| KfKm – DfKm | 0.6328 |


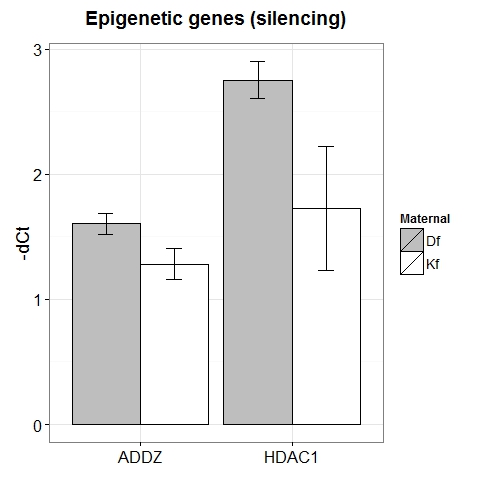

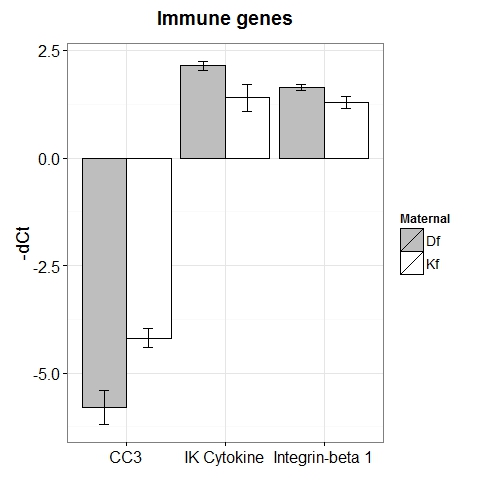


Fig. S1 Maternal effect on relative gene expression (–δCt ). From left to right: Epigenetic genes silencing (ADDZ, HDAC1), immune genes (CC3, IK Cytokine, Integrin-beta 1). Danish female in grey, Kiel female in white. Bar charts with standard error.


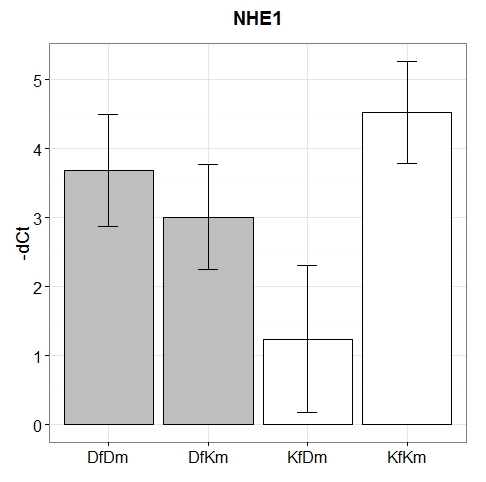

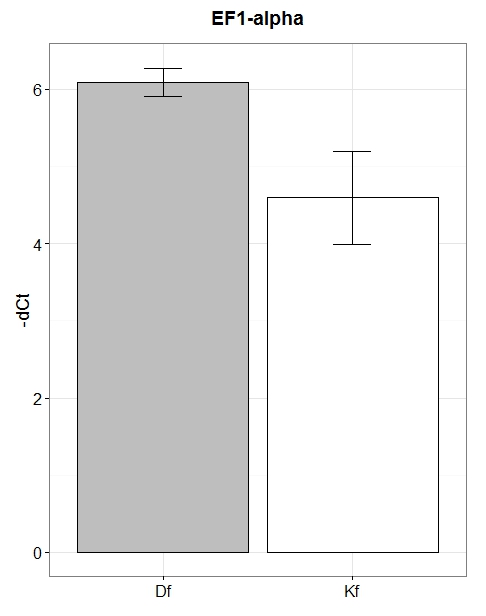


Fig. S2 Parental effect on relative gene expression (–δCt ). From left to right: Maternal effect on EF1-alpha (Cell function gene), interaction of maternal and paternal origin on NHE1 (osmoregulation gene). Danish female in grey, Kiel female in white. Bar charts with standard error.


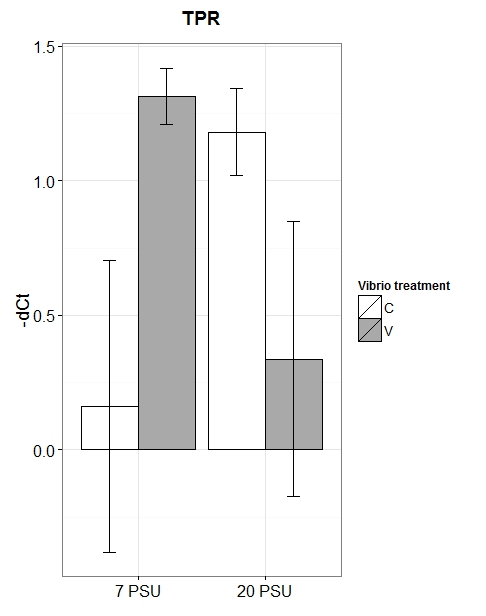


Fig. S3 Interaction of Salinity and *Vibrio* on relative gene expression (–δCt ). TPR gene (epigenetic gene - activation). Vibrio treatment in grey, control in white. Bar charts with standard error.
